# Immunological biomarkers at birth and later risk of celiac disease

**DOI:** 10.1186/s12876-025-03743-z

**Published:** 2025-03-11

**Authors:** Maria Ulnes, Veroniqa Lundbäck, Susanne Lindgren, Mattias Molin, Rolf H. Zetterström, Olov Ekwall, Karl Mårild

**Affiliations:** 1 Department of Pediatrics, Queen Silvia Children’s Hospital, Gothenburg, Sweden; 2 Department of Pediatrics, Institute of Clinical Sciences, Sahlgrenska Academy, University of Gothenburg, Gothenburg, Sweden; 3 Centre for Inherited Metabolic Diseases, Karolinska University Hospital, Stockholm, Sweden; 4 Department of Molecular Medicine and Surgery, Karolinska Institute, Stockholm, Sweden; 5 Department of Rheumatology and Inflammation Research, Sahlgrenska Academy, University of Gothenburg, Gothenburg, Sweden; 6 Statistiska konsultgruppen, Gothenburg, Sweden

**Keywords:** TREC, KREC, Epigenetic cell counting, Dried blood spots, Newborn, Celiac disease

## Abstract

**Background:**

The role of immune cell profiles at birth in determining the risk of celiac disease (CD) development is currently unestablished. This study aimed to determine the associations between T- and B-cell profiles at birth and pediatric CD.

**Methods:**

This regional cohort study analyzed prospectively collected dried blood spots from 158 children with CD (median 7 years old at CD diagnosis) and two matched comparators each (*n* = 316). We quantified T-cell receptor excision circles (TRECs) and kappa-deleting recombination excision circles (KRECs) as measures of thymic and bone marrow output at birth. Moreover, we used epigenetic cell counting to estimate the percentages of lymphocyte subsets: CD3+, CD4+, CD8 + T cells, CD4 + memory T, regulatory T, B, and NK cells.

**Results:**

No associations were found between measured immune cell markers at birth and CD development (all p values > 0.26). The median number of copies was 120 for TRECs (IQR = 92–168) and 136 (IQR = 91–183) for CD patients and comparators, respectively, and for KRECs, it was 69 (IQR = 45–100) for CD patients and 66 for comparators (IQR = 44–93). Across the groups, there were similar median percentages of T cells (CD, 32.6% [IQR = 27.0–43.8%] vs. comparators, 33.9% [IQR = 26.3–45.7%]) and B cells (CD, 25.4% [IQR = 20.3–30.6%] vs. comparators, 24.7% [IQR = 19.9–30.8%]). The ratio of the lymphocyte subset estimates between CD patients and comparators approximated one; all p values were > 0.26. The results were consistent across strata defined by sex, HLA type, and age at diagnosis.

**Conclusion:**

Genetic and epigenetic markers for B cells and T cells in immune cell profiles at birth did not impact susceptibility to childhood-onset CD.

**Supplementary Information:**

The online version contains supplementary material available at 10.1186/s12876-025-03743-z.

## Background

Celiac disease (CD) is a gluten-driven enteropathy, caused by a dysregulated immune response in genetically susceptible individuals [1]. Early life environmental factors contribute to the disease development [1–3]. The potential effect of such exposures is likely mediated by changes in the developing immune system, but the precise nature and timing of such events are currently unestablished [4]. 

Autoreactive T- and B cells play a central role in the pathogenesis of CD [1, 5]. In essence, T cells mediate the immune response by recognizing the gluten peptides presented on the humane leucocyte antigen (HLA) molecules, and B cells contribute by producing CD-specific antibodies [5]. Observations from patients with inborn errors of immunity (with extremely low, or absent central output of T- and/or B cells) suggest that significantly altered T- and B-cell output at birth may influence the susceptibility to autoimmunity [6]. 

T-cell receptor excision circles (TRECs) and kappa-deleting recombination excision circles (KRECs) are byproducts of T- and B-cell development, that reflect the T- and B-cell output from the thymus and bone marrow, respectively (supplemental digital content, SDC, Figure S1) [7]. These extrachromosomal DNA fragments can be approximated from dried blood spots (DBS) and are increasingly used in newborn screening, where absent or very low levels of TRECs or KRECs signal inborn errors of immunity [8–10]. 

The gold standard for approximating lymphocytes in peripheral blood is flow cytometry, which requires fresh blood. Taking advantage of cell-type specific signatures, epigenetic cell counting can be used for quantification of immune cell subsets from dried blood spots, which correlates well with flow cytometry [11]. 

Patients with severely altered development of T and B lymphocytes due to inborn errors of immunity are at increased risk of developing autoimmune diseases [6, 12]. Observations from other pediatric autoimmune diseases indicate that TRECs are reduced in manifest juvenile idiopathic arthritis (JIA) and immune thrombocytopenia [13, 14], elevated in type 1 diabetes [15], but normal at birth, long before JIA onset [16]. It remains unknown whether normal variation in immune cell profiles at birth influences the risk of childhood CD.

This regional cohort study applied novel qPCR assays to prospectively collected dried blood spots (DBS) and examined whether measures of B- and T-cell profiles at birth predicted later CD development.

## Methods

Using qPCR on prospectively collected dried blood spot (DBS) samples from newborn screening, we examined the levels of TREC and KREC and the relative proportions of B- and T-cell subpopulations at birth in 158 children who later developed CD vs. 1–2 comparators, matched by sex, gestational age, and date of birth.

### Study design, setting, and participants

This study was based on 158 children from a regional cohort from Queen Silvia Children’s Hospital (Gothenburg, Sweden) [17]. The children were aged between 8 and 18 years at study enrollment and were diagnosed according to European guidelines for CD in children [18]. Only children born in Sweden, hence eligible for newborn screening, were included.

### Medical record data

By reviewing patients’ charts alongside study enrollment in the first six months of 2021, we retrieved information on the diagnosis of CD (non-biopsy/biopsy approach, including Marsh classification [19]). We also retrieved information on the date of diagnosis (defined as the time of biopsy or, in instances of a non-biopsy approach to CD diagnosis [18], the time when the diagnosis was communicated to the patient). We retrieved additional data on their HLA haplotype and classified them into high and low genetic risk. The high-risk group included individuals homozygous for high-risk alleles (encoding HLA-DQ2.5 or HLA-DQ2.2), as well as those carrying a combination of these (HLA-DQ2.5 *and* HLA-DQ2.2), while other combinations were classified as low-risk (individuals heterozygous for HLA-DQ2.5, HLA-DQ2.2, or HLA-DQ8).

### Questionnaire data

We used an electronic form to collect parent-reported data on prenatal and perinatal events as well as medical history up to study enrollment. These data are summarized in Table 1 and Supplementary Tables S1-S2. Any questionnaire data were available for 153 patients (96%). Pre- and perinatal data collected included maternal use of medicines during pregnancy, smoking, and nicotine usage during pregnancy. We also collected data on common maternal complications during pregnancy (e.g., hypertension or preeclampsia and other severe diseases), infections, and the use of antibiotics. The data collected on perinatal events included parity, mode of delivery, and the need for neonatal care. We have previously presented data on the children’s medical history and hereditary factors [17]. 

**Table 1:** Characteristics of children with Celiac disease (CD) and matched comparators

	CD cases (*n* = 158)	Comparators (*n* = 316)
**Matching criteria, data from the phenylketonuria (PKU) biobank**		
***Age***^***1***^, *years*,* median*,* (IQR)*	13 (11; 16)	13 (11; 16)
***Girls***, *n (%)*	92 (58%)	184 (58%)
***Gestational age***, *weeks*, *median*,* (IQR)*	40 (39; 41)	40 (39; 41)
**Data on CD diagnosis**, medical record data*		
***Age at CD diagnosis***, *median*,* (IQR)*	7 (5; 9)	
***Biopsy-verified CD diagnosis***, *n (%)*	63 (40)	
***HLA-type***, *n (%)*		
*HLADQ2.5/DQ2.5*	36 (23)	
*HLADQ2.2/DQ2.2*	2 (1)	
*HLADQ2.5/DQ2.2*	20 (13)	
*HLADQ2.5/X*,* DQ2.2/X*,* DQ8/X*	93 (59)	
*HLADQ8/8*	7 (4)	
**Patient background**, questionnaire data*		
***Comorbidity***, *n (%)*		
*Thyroid disease*	4 (3)	
*T1DM*	4 (3)	
***Heredity (first-degree relative)***, *n (%)*		
*CD*	36 (27)	
*Thyroid disease/Type 1 diabetes*	30 (19)	
**Preand perinatal data**, questionnaire data*		
***Cesarean section***, *n (%)*	25 (16)	
***Admission to neonatal ward***, *n (%)*	8 (5)	
***Mother’s conditions during pregnancy***, *n (%)*		
*Infectious disease* ^*2*^	20 (13)	
*Other conditions* ^*3*^	5 (3)	
***Mother’s use of medication during pregnancy***, *n (%)*		
*Antibiotics*,* corticosteroids*,* vaccines*	19 (12)	
*Others* ^*4*^	76 (8)	
***Mother’s use of nicotine during pregnancy***, *n (%)*	7 (5)	

*Based on available data from the PKU biobank (158 CD cases and 316 comparators), medical records (158 CD cases), and questionnaires (153 CD cases). Data from medical records were not available for comparators due to ethical approval

^1^ Sample retrieval from the biobank

^2^ Gastroenteritis, urinary tract infection, pneumonia, and other infections treated with antibiotics

^3^ Conditions not requiring antibiotics (e.g., preeclampsia)

4 Including antidepressants, anxiolytics, analgesics, hormones, antihypertensives, and other medications

HLA, human leukocyte antigen; IQR = interquartile range

### Data from the PKU biobank

In adherence to the Swedish newborn screening protocol, a venous blood sample is obtained promptly after 48 h of age (48–72 h for most children). Four blood samples were collected on filter paper, air dried and later stored along with the relevant sample details. The national phenylketonuria (PKU) biobank for newborn screening (Karolinska University Hospital, Stockholm, Sweden) preserves DBS samples obtained from nearly all newborn screenings since January 1, 1975 [20]. We collected data from the PKU biobank on the matching criteria of gestational age at birth (week), sex, and date of birth.

### Sample formation

Hence, we searched the PKU biobank for DBS cards of 162 children with CD in the above-described cohort. The samples were prospectively collected at birth between 2003 and 2013 and were accessed shortly before the laboratory analyses began in May and June 2022. Two DBS cards (1.2%) were not available in the biobank. The remaining 160 CD cases were identified in the biobank and matched for sex, gestational age (week), and date of birth with two comparators (*n* = 320). We used four punches of 3.2 mm from the DBS cards per individual, one for TREC/KREC analysis and three for epigenetic cell counting. We did not repeat analyses that were deemed unsuccessful to avoid excess use of samples in the biobank.

Two comparators were employed for the TREC and KREC analyses, whereas limited test material availability allowed only one comparator per case in the epigenetic cell counting analyses.

**Fig. 1:**
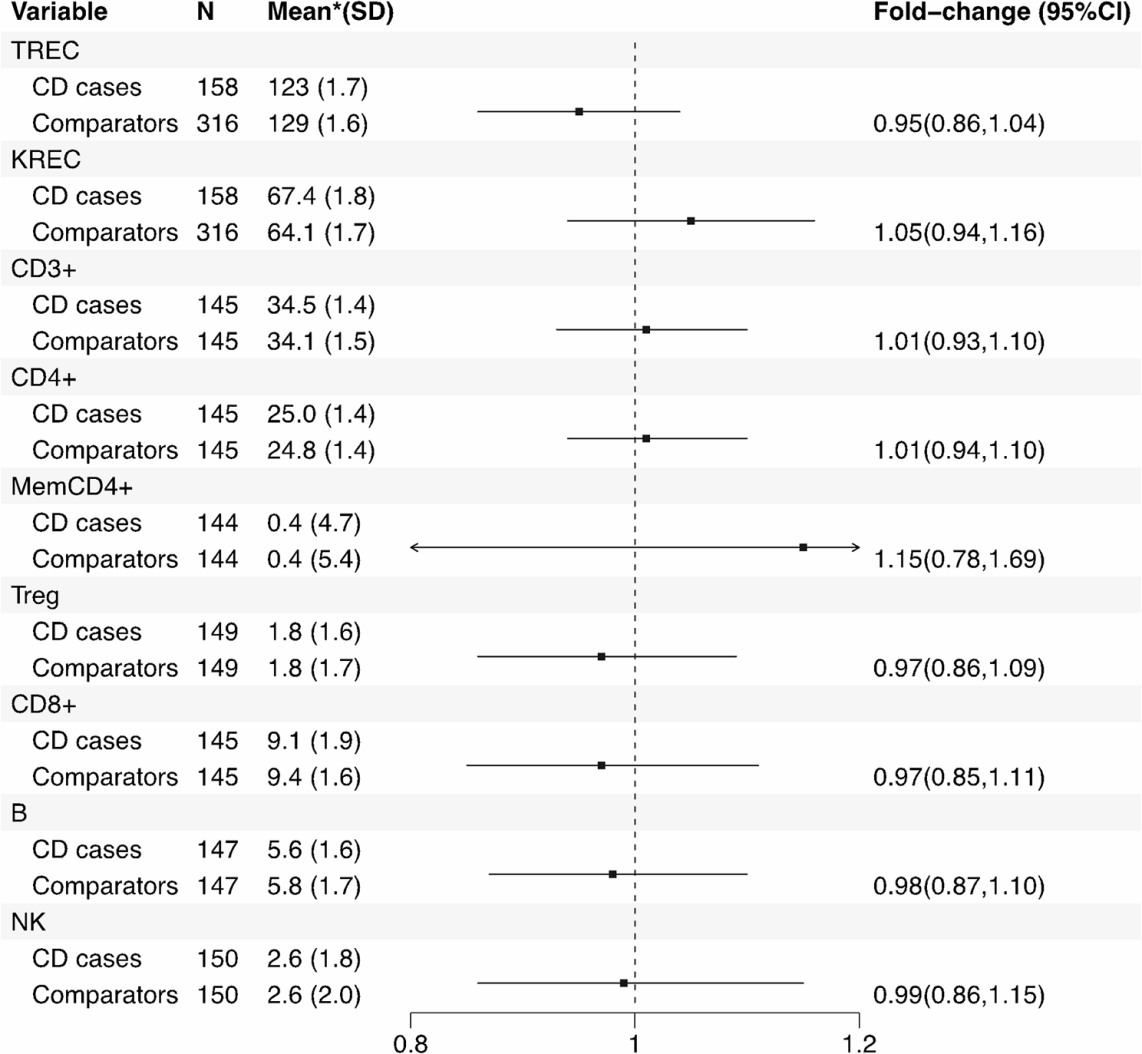
Association of fold change in immune cell profiles at birth with later celiac disease (CD). Forest plot of the fold change in immune cell profiles at birth in celiac children (CD cases) and comparators: TREC and KREC levels (measures of T- and B-cell output), expressed as the number of copies/punch, and lymphocyte subsets, expressed as the percentage of nucleated cells in the sample: overall T cells (CD3+), T-helper cells (CD3+/CD4+), cytotoxic T cells (CD3+/CD8+), memory (CD4+/CD45 RO+), FOX PR/TSDR+ regulatory (Treg) T cells, B cells (CD19+) and NK cells (CD16+/CD56dim). The variables were log-transformed before analysis. The fold change refers to the ratio between CD patients and comparators *Geometric mean CI = confidence interval, KREC = kappa-deleting recombination excision circle SD=Standard deviation, TREC = T-cell receptor excision circle

Following exclusion due to failed qPCR, we assessed data from 158 cases and 316 comparators (98.8%) for KREC and TREC, as well as up to 150 CD cases and 150 comparators (93.7%) for epigenetic cell counting. (Exact numbers are displayed in the flowchart in Supplementary Figure S2 and Fig. 1).

### Markers of immune cell profiles

Using the protocol described by Göngrich et al. [10], we analyzed TREC, KREC, and beta-actin (ACTB) copies in 3.2 mm punches of DBS screening cards. The SPOTit-TK kit in a 96-well format (ImmunoIVD, Nacka, Sweden) was originally intended for the use of DBS from newborn screening. On each 96-well plate, three internal DBS controls (low-TREC/high-KREC, low-KREC/high-TREC, and low-TREC/low-KREC) and one blank PCR (no DBS) were analyzed along with samples from 26 CD cases and 2 controls/cases. MiniAmp Thermal Cyclers (Thermo Fisher Scientific, Waltham, MA, USA) was used for DNA delusion, and the real-time PCR was run in Applied Biosystems QuantStudio 5 Dx instruments (Thermo Fisher Scientific, Waltham, MA, USA) with the QuantStudio 5 Dx IVD Software v1.0. We used 5-point standard curves (provided in the kit) ranging from 10 to 100,000 copies/well for TREC and KREC quantification and from 100 to 1,000,000 copies/well for ACTB.

To determine the proportions of specific T- and B-cell subpopulations, we also performed epigenetic cell counting using the same DBS cards. The iMune TBNK assay (Epimune GmbH, Berlin, Germany), modified with regulatory T (Treg) cells as additional targets, was used [9, 11, 21]. The DNA was bisulfite-converted and purified, followed by the amplification of bisulfite-converted target DNA via real-time PCR. Cell type-specific demethylated genomic regions for the following cell types were targeted: Nucleated cells (GAPDH), overall T cells (CD3+), T-helper cells (CD3+/CD4+), memory T cells (CD4+/CD45 RO+), cytotoxic T cells (CD3+/CD8+), Tregs (FOXP3), B cells (CD19+) and NK cells (CD16+/CD56dim) [11, 21]. (The lymphocyte subsets are illustrated in Supplementary Figure S1) The result for each lymphocyte subset is weighed against the amplification of unmethylated glyceraldehyde 3-phosphate dehydrogenase (GAPDH), which is present in all nucleated cells. Thus, subsets of lymphocytes are approximated as a percentage of nucleated cells in the sample [11]. We used an Applied Biosystems QuantStudio 5 Dx instrument (Thermo Fisher Scientific, Waltham, MA, USA) using the QuantStudio 5 Dx IVD Software v1.0, with a 96-well plate to amplify target cell-specific demethylated regions in the bisulfite-converted DNA. Each 96-well plate accommodated the samples from four CD cases and their four comparators (one/CD case). Two positive and one negative control (iMune Check, IM-CHECK-01) supplied by the kit manufacturer were included with every run, and in each PCR well, internal control was co-amplified to guarantee the quality, sample preparation, and adequate DNA concentration. According to the manufacturer, the lower limit of quantitation for each assay was 80 copies for each cell type. We did not repeat qPCR due to the limited test material. Quality assurance analyses were performed according to the manufacturer’s instructions.

### Statistical analyses

Each CD patient was paired with two comparators from the PKU biobank for KREC and TREC and with one comparator for epigenetic cell counting. We analyzed the associations between later CD and the number of copies/stance (TREC and KREC) and estimated subpopulations of lymphocytes (CD3+, CD3+/CD4+, CD4+/CD45 RO+, CD3+/FOXP3, CD3+/CD8+, CD19+, and CD16+/CD56dim), approximated as percentages of nucleated cells in the sample. We did not exclude outliers because epigenetic cell counting is a new technique without predefined ranges for newborn DBSs. Analyses were limited to complete matching sets for all immune cell variables. Missing data (0.2% for KREC and TREC and 6.3% for epigenetic cell counting) were mainly related to unsuccessful analysis.

Owing to the nonnormal distribution of the assayed variables, we calculated the median and interquartile range (IQR), while logarithmically transformed values were used to calculate the geometric mean and standard deviation (SD). The logarithmically transformed values were also used for between-group comparisons via Student’s t-test, fold changes, and 95% confidence intervals (CIs) for the ratios of measured immune markers between CD patients and matched comparators. To test for different group effects of sex and age at CD diagnosis (< 7 or ≥ 7 years) on immune cell biomarkers, analysis of variance (ANOVA) was used together with an interaction effect. Spearman rank correlation was used to test for correlation between KRECs and B cells, and TREC and T cells. The statistical analyses were performed using IBM SPSS Statistics (v29.0.1.1) and SAS version 9.4 (SAS Institute, Cary, NC, USA). Figures were produced via the R (R Core Team, 2023, Vienna, Austria) package ggplot2 (Wickham, 2009).

### Ethical approval

This study was approved by the Swedish Ethical Review Authority (reference number 2020–06033) and was carried out in accordance with the Code of Ethics of the World Medical Association (Declaration of Helsinki). Upon study inclusion, written informed consent was obtained from the study participants and parents. The ethical permit did not allow us to contact and collect data on the comparators’ HLA haplotype and medical history [22]. 

## Results

Among the 158 celiac children and 316 comparators included in this study; the majority were girls (58%; Table 1). Seven of the celiac children (4%) were born preterm (at 34–36 weeks of gestation). The median age at CD diagnosis was seven years (IQR 5; 9), and most children were diagnosed via a non-biopsy approach (*n* = 95, 60%). In Supplementary Tables S1-S2, background characteristics are displayed stratified by sex and age at diagnosis.

### Similar immune cell profiles at birth in CD patients and comparators

**Fig. 2:**
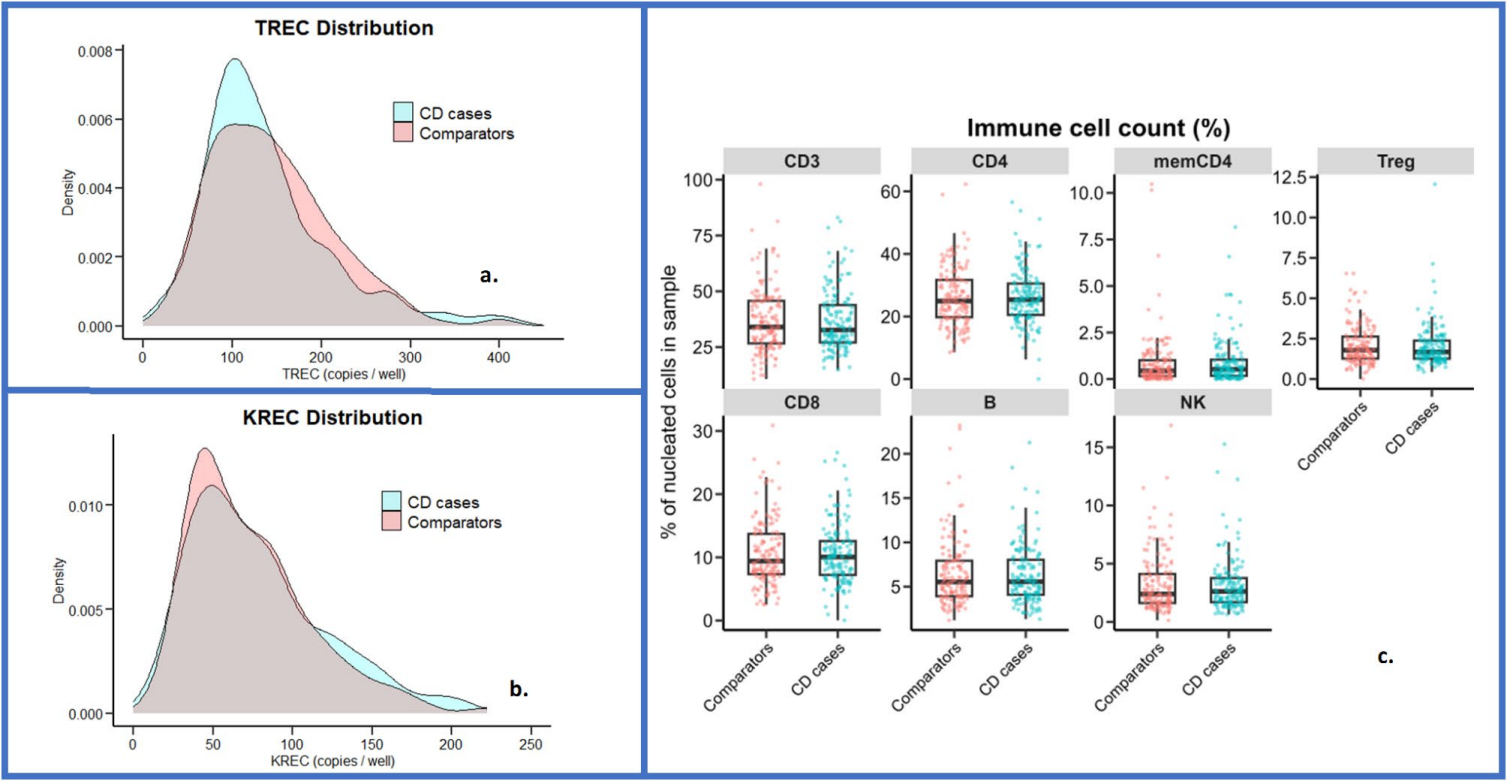
Distribution of TREC and KREC and multipaneled boxplot of lymphocyte subsets at birth in children with later celiac disease (CD) and matched comparators. **a-b.** Density plots of T-cell receptor excision circles (TRECs) and kappa-deleting recombination excision circles (KRECs) at birth in 158 CD patients (blue) and 316 comparators (red), expressed as the number of copies/3.2 mm punch. **c.** Multipaneled boxplot of lymphocyte subsets at birth in up to 150 CD cases (right; blue) and up to 150 comparators (left, red), estimated by epigenetic cell counting and expressed as % of nucleated cells in the sample: overall T cells (CD3+), T-helper cells (CD3+/CD4+), cytotoxic T cells (CD3+/CD8+), memory (CD4+/CD45 RO+), FOX PR/TSDR+ regulatory T cells, B cells (CD19+), and NK cells (CD16+/CD56dim). Boxes display median, upper, and lower quartiles, and whiskers extend to 1.5 × the interquartile range

At birth, children who developed CD had similar distributions of TREC (T-cell output), KREC (B-cell output), and lymphocyte subset percentages as the matched comparators (Fig. 2; Table 2). All the children had TREC and KREC levels above the cutoff for newborn screening for severe primary immunodeficiencies [10]. The median number of TREC copies was 120 (IQR = 92–168) in CD patients and 136 (IQR = 91–183) in comparators (*p* = 0.18). The median number of KREC copies was 69 (IQR = 45–100) in CD patients and 66 in comparators (IQR = 44–93, p-value = 0.38 [Table 2]).

Similarly, the median proportions of T cells and B cells were consistent across both groups (Fig. 2; Table 2). The median percentage of CD3 + cells was 32.6% (IQR = 27.0-43.8%) in CD cases and 33.9% (IQR = 26.3–45.7%) in comparators (p-value = 0.35). The median percentage of CD19 + cells was 25.4% (IQR = 20.3–30.6%) in CD cases and 24.7% (IQR = 19.9–30.8%) in comparators (*p* = 0.87 [Table 2]). The fold-change estimates of the measured immune cell profiles and CD risk approximated one, ranging from 0.95 to 1.15 (all p-values > 0.26) (Fig. 1). TRECs and KRECs correlated with T cells and B cells assessed by epigenetic cell counting, respectively (for correlation between TRECs and CD3 + T cells, r-value 0.31; for correlation between KREC and B cells, r-value 0.66, both p-values < 0.001, Supplementary Figures S3 and S4). Finally, stratified analyses revealed no discernible differences in outcomes based on sex, age at diagnosis, or HLA haplotypes (Supplementary Tables S3-S5).

**Table 2:** Immune cell profiles at birth of children with Celiac disease (CD) and matched comparators

	CD cases Median, IQR	Comparators Median, IQR	*p*-value
**Markers of T- and B- lymphocyte output**			
*n observations*	158	316	
**TRECs**, no. of copies	120 (92; 168)	136 (44; 184)	0.27
**KRECs**, no. of copies	69 (45; 100)	66 (44; 93)	0.39
**Epigenetic cell counting**,** lymphocyte subset proportions (of total leukocyte population)**			
n observations*	*n* = 150	*n* = 150	
**CD3+**, %	32.6 (27.0; 43.8)	33.9 (26.3; 45.7)	0.79
**CD4+**, %	25.4 (20.3; 30.6)	24.7 (19.7; 31.8)	0.73
**memCD4+**, %	0.52 (0.17; 1.0)	0.43 (0.15; 1.0)	0.48
**Treg**, %	1.7 (1.3; 2.4)	1.8 (1.2; 2.7)	0.61
**CD8+**, %	9.9 (7.1; 12.5)	9.4 (7.2; 13.8)	0.66
**B**, %	5.6 (4.1; 8.1)	5.7 (4.0; 7.9)	0.68
**NK**, %	2.6 (1.7; 3.8)	2.4 (1.6; 4.2)	0.92

T-cell receptor excision circle (TREC) and kappa-deleting recombination excision circle (KREC) levels at birth, expressed as the number of copies/punch and lymphocyte subsets, are expressed as the percentage of nucleated cells in the sample: overall T cells (CD3+), T helper cells (CD3+/CD4+), cytotoxic T cells (CD3+/CD8+), memory (CD4+/CD45 RO+), FOX PR/TSDR + regulatory (Treg) T cells (CD19+) and NK cells (CD16+/CD56dim) in children who developed CD and matched comparators. Measures of dispersion are based on nontransformed values

*The number of observations for each subset is displayed in Fig. 1

IQR = Interquartile range

## Discussion

By quantifying genetic and epigenetic markers of T cells and B cells, this first study found no evidence that immune cell profiles at birth significantly influence the risk of CD in children.

Given the pivotal role of autoreactive T cells and the significant involvement of B cells in CD [1], understanding the mechanisms of central tolerance is crucial for comprehending disease development. In this context, newborn screening data may provide valuable insights, particularly through TREC and KREC levels, which reflect the central outputs of T cells and B cells from the thymus and bone marrow, respectively. Insights from inborn errors of immunity, with low or absent TREC or KREC levels at birth, suggest that significantly reduced central output is linked to an increased risk of developing autoimmune diseases, including CD [6]. Such reduced central output could result from an inadequate interaction between hematopoietic and stromal cells, disrupting central tolerance induction. Thus, an increased proportion of self-reactive cells may reach the periphery and give rise to autoimmunity [6, 23]. In addition, under lymphopenic conditions, homeostatic T-cell proliferation may lead to the expansion of self-reactive clones, resulting in autoimmune diseases [24, 25]. 

While the associations between TREC and KREC and established CD have not been examined, low levels of TREC, but not KREC, have been observed in pediatric patients diagnosed with other autoimmune conditions, such as JIA and ITP [13, 14]. In contrast, type 1 diabetes has been associated with elevated TREC (but not KREC) levels [15]. Our null findings are consistent with a recent report of normal thymic and bone marrow function at birth in children who later developed JIA [16]. 

Environmental factors, including infections in early life, may shape the risk of later CD [3], although the timing and mechanisms through which these factors interact with the immune system remain unclear [2]. Studies involving children genetically predisposed to CD have identified physiological changes that may precede the onset of the disease [26–28]. 

In children with a genetic risk for CD, recent findings have identified specific plasma lipid and phospholipid patterns in the first three months of life in infants who later developed CD [26–28]. Phospholipids have been implicated in regulating T-cell immunity and are thus potentially important in CD [27]. Additionally, certain cytokine patterns are distinguishable in four-month-old infants who later develop CD [28], suggesting that immunological discrepancies may arise very early in life. Given that such phospholipid and cytokine patterns have not been distinguished at birth [26, 29], underscores the need for further investigations into the perinatal period as a critical window for biological changes. The strongest genetic risk factor for CD is HLA [5]. We found no differences in TRECs, KRECs, or immune cells at birth according to CD-associated HLA haplotypes.

In the PROFICEL study of children at genetic risk for CD, lymphocyte subsets at four months of age were associated with environmental triggers, including infections, diet, antibiotic use, and gut microbiome composition. However, it remains unclear whether these lymphocyte subsets also influence the later risk of CD [4]. Specifically, in their study, infections during the first months were linked to an overall increase in the number of CD4 + T cells, activated CD4+ (CD3 + CD4 + CD45RO+) Tregs (CD4 + CD25+), and NK cells, suggesting that certain infections may induce protective immune activation, stimulating immune tolerance [3, 4]. Although the specific link between lymphocyte subsets in infancy and later CD development remains to be fully understood, our findings did not reveal such an association at birth.

Our study is seemingly the first to examine the potential influence of immune cell profiles at birth on the risk for the development of CD. The scarcity of similar studies is likely related to difficulties in obtaining adequate samples for flow cytometry, given the limited availability of newborns’ blood. Our use of epigenetic cell counting on DBSs collected at birth provides new possibilities for exploring lymphocyte subsets during this critical period in life. During the first three months in life, T-cell percentages start high and decrease [30, 31], emphasizing their critical role in early immune defense, while B-cell percentages begin lower and gradually increase as the adaptive immune defense matures [30, 31]. By using epigenetic cell counting we found lymphocyte subsets that were comparable to flow cytometry data of healthy newborns [30, 31]. As CD3 + includes all peripheral T cells, and TRECs reflect recent thymic emigrants, analysis of demethylated CD3 + copies corresponds better to absolute T cell counts measured by flow cytometry than TRECs [9]. The weak correlation between TRECs and demethylated CD3 + copies in our material (*r* = 0.31) matches data from flow cytometry in a newborn screening setting (TRECs from DBS and CD3+% by flow cytometry [*r* = 0.31, *p* < 0.001]) [32]. For KRECs, a moderate correlation was observed with B cell percentage (*r* = 0.66), similar to that between KRECs and B cells, measured by flow cytometry(*r* = 0.42, both *p* < 0.001) [32]. However, the subsets of T cells (CD3+), T-helper cells (CD3+/CD4+), CD4 + memory T cells (CD4+/CD45 RO+), cytotoxic T cells (CD3+/CD8+), regulatory T cells (Treg; FOXP3+), B cells (CD19+) and NK cells (CD16+/CD56dim) at birth do not predict the future development of CD in the child. Our null findings argue against a significant influence on CD risk, or clinical course by T- and B-cell subsets, as well as NK cells at birth.

The strengths of this study include our use of a novel assay for determining lymphocyte subsets in DBSs, enabling the analysis of prospectively collected peripheral blood samples. We applied a validated technique for quantifying TRECs and KRECs. Additionally, our relatively large and well-defined sample of children with CD [17], along with a matched design accounting for perinatal factors, such as sex and gestational age, which affect TREC and KREC levels, adds robustness to our findings.

However, this study has several limitations. Due to the ethical approval for this study, we were unable to collect data on comparators’ HLA type and medical history, which prevented us from accounting for these factors [22]. The risk of CD in Sweden has been reported to be 1.8% [33]. While we therefore cannot rule out that individual comparators may have been diagnosed with CD, they should be limited and unlikely to significantly influence our results. Nevertheless, the consistency of our results argues against negative residual confounding as the sole explanation for our null findings. Furthermore, the median age at diagnosis in our study was seven years, and the extent to which our findings can be generalized to children with very early-onset CD [34] is unknown. Finally, while this study examined the quantity and proportion of selected immune cells, the possibility that alterations in cell function may influence subsequent CD risk cannot be ignored.

In conclusion, our findings indicate that immune cell profiles at birth have no significant influence on the subsequent risk of childhood-onset CD.

## Electronic supplementary material

Below is the link to the electronic supplementary material.

Supplementary Material 1

## Data Availability

The data collected for this article will be shared upon reasonable request to the study’s PI, Karl Mårild (karlmarild@gmail.com).

## Abbreviations

ACTB: beta-actin/actin beta
CD: celiac disease
DBS: dried blood spot
GAPDH: glyceraldehyde 3-phosphate dehydrogenase
HLA: human leukocyte antigen
IQR: interquartile range
JIA: juvenile idiopathic arthritis
KREC: kappa-deleting recombination excision circle
PKU: phenylketonuria
qPCR: quantitative polymerase chain reaction
SD: standard deviation
TREC: T-cell receptor excision circle
