# Supplementary material for: Immunological biomarkers at birth and later risk of celiac disease
Source: BMC Gastroenterol. 2025 Mar 11;25:159. doi: 10.1186/s12876-025-03743-z (PMC11899458; doi:10.1186/s12876-025-03743-z)
Supplement: Supplementary file 1 — Supplementary Material 1 [file 12876_2025_3743_MOESM1_ESM.docx]

# **SUPPLEMENTARY MATERIAL**

**Immunological biomarkers at birth and later risk of celiac disease**

**Authors**
**Maria Ulnes**, Veroniqa Lundbäck, Susanne Lindgren, Mattias Molin, Rolf H. Zetterström, Olov Ekwall, Karl Mårild

TABLE OF CONTENTS

[SUPPLEMENTARY MATERIAL 1](#_Toc188258883)

[**ABBREVIATIONS** 2](#_Toc188258884)

[**SUPPLEMENTARY METHODS** 3](#_Toc188258885)

[*Power analysis* 3](#_Toc188258886)

[**SUPPLEMENTARY TABLES** 4](#_Toc188258887)

[**Table S1.** Characteristics of children with celiac disease (CD), stratified by sex. 4](#_Toc188258888)

[**Table S2.** Characteristics of children with celiac disease (CD), stratified by age at diagnosis. 5](#_Toc188258889)

[**Table S3.** Association between immune cell profiles at birth in children with celiac disease (CD) and matched comparators, stratified analyses by sex. 6](#_Toc188258890)

[**Table S4.** Association between immune cell profiles at birth in children with celiac disease (CD) and matched comparators, stratified analyses by age at diagnosis (≥7 years or younger). 7](#_Toc188258891)

[**Table S5.** Association between immune cell profiles at birth in children with celiac disease (CD), presented for carriers of two high risk alleles (HLA DQ 2.5 and/or HLA DQ 2.2) vs. one of these alleles, or HLA DQ 8. 8](#_Toc188258892)

[**FIGURES** 9](#_Toc188258893)

[**Figure S1** Schematic overview of the measured immune cell markers in peripheral blood. 9](#_Toc188258894)

[**Figure S2** Flowchart of the formation of the study sample 11](#_Toc188258895)

[**Figure S3** Correlation between TRECs and CD3% 12](#_Toc188258896)

[**Figure S4** Correlation between KRECs and B cells 13](#_Toc188258897)

[**REFERENCES** 14](#_Toc188258898)

## **ABBREVIATIONS**

CD=Celiac disease

DBS=Dried blood spot

HLA=Human leukocyte antigen

GAPDH=Glyceraldehyde 3-phosphate dehydrogenase

KREC= Kappa-deleting recombination excision circle

PKU=phenylketonuria

TREC=T-cell receptor excision circle

## **SUPPLEMENTARY METHODS**

### *Power analysis*

In another study on TREC and KREC measurements on neonatal DBS from the same biobank, the mean TREC value was 86 copies/punch (SD 43), and the mean KREC value was 61 (35) copies/punch in healthy comparators. (1) Given a two-sided 5% significance level, our *a priori* power calculation indicated 80% power to detect a mean difference in TREC of at least 12 copies/punch for 162 CD cases and 324 matched comparators. The corresponding minimum detectable mean difference for KREC analyses was 10 copies/punch.

## **SUPPLEMENTARY TABLES**

### **Table S1.** Characteristics of children with celiac disease (CD), stratified by sex.

|  | **Girls**  (n=92) | **Boys**  (n=66) |
| --- | --- | --- |
| **Matching criteria, data from the phenylketonuria (PKU) biobank** | | |
| ***Age^1^,*** *years, median, (IQR)* | 13 (12.; 16) | 13 (11; 16) |
| ***Gestational age,*** *weeks,* *median, (IQR)* | 40 (39; 41) | 40 (39; 41) |
| **Data on CD diagnosis, from medical records** | | |
| ***Age at CD diagnosis,*** *median, (IQR)* | 7 (6; 9) | 8 (5; 9) |
| ***Biopsy-verified CD diagnosis,*** *n (%)* | 37 (40) | 26 (40) |
| ***HLA-type,*** *n (%)* |  |  |
| *HLADQ2.5/DQ2.5* | 21 (23) | 15 (22) |
| *HLADQ2.2/DQ2.2* | 1 (1) | 1 (2) |
| *HLADQ2.5/DQ2.2* | 11 (12) | 9 (14) |
| *HLADQ2.5/X, DQ2.2/X, DQ8/X* | 55 (60) | 38 (58) |
| *HLADQ8/8* | 4 (4) | 3 (5) |
| **Patient background, data from questionnaire** | | |
| ***Comorbidity****, n (%)* |  |  |
| *Thyroid disease* | 2 (2) | 2 (3) |
| *Type 1 diabetes* | 2 (2) | 2 (3) |
| ***Heredity (first-degree relative),*** *n (%)* |  |  |
| *CD* | 18 (23) | 18 (33) |
| *Thyroid disease/Type 1 diabetes* | 17 (19) | 13 (19) |
| **Pre- and perinatal data, from questionnaire** |  |  |
| ***Cesarean section,*** *n (%)* | 15 (16) | 10 (15) |
| ***Admission to neonatal ward****, n (%)* | 4 (5) | 4 (7) |
| ***Mother’s conditions during pregnancy,*** *n (%)* |  |  |
| *Infectious disease^2^* | 12 (13) | 8 (12) |
| *Other conditions^3^* | 5 (4) | 0 |
| ***Mother’s use of medication during pregnancy,*** *n (%)* | | |
| *Antibiotics, corticosteroids, vaccines* | 11 (5) | 8 (9) |
| *Others^4^* | 44 (48) | 32 (49) |
| ***Mother’s use of nicotine during pregnancy****, n (%)* | 4 (5) | 3 (5) |
|  |  |  |

Data from the Swedish PKU biobank, patient medical records, and questionnaires, displayed for CD cases stratified on sex. Data from medical records were not available in comparators due to ethical approvals.

Percentages are reported from available data. *Any* questionnaire data was available for n=153 CD cases (96%).

^1^ At sample retrieval from the PKU biobank

^2^ Gastroenteritis, urinary tract infection, pneumonia, and other infections treated with antibiotics.

^3^ Conditions not requiring antibiotics (e.g., preeclampsia)

^4^ Including antidepressants, anxiolytics, analgesics, hormones, antihypertensives, and other medications.

HLA= human leukocyte antigen, IQR= interquartile range

### **Table S2.** Characteristics of children with celiac disease (CD), stratified by age at diagnosis.

|  | **<7 years**  n=72 | **≥7 years**  n=86 |
| --- | --- | --- |
| **Matching criteria, data from the phenylketonuria (PKU) biobank** | | |
| ***Age^1^,*** *years, median, (IQR)* | 12 (10; 13) | 16 (13; 17) |
| ***Sex,*** *girls* *n (%)* | 42 (58) | 50 (59) |
| ***Gestational age,*** *weeks,* *median, (IQR)* | 40 (39-41) | 40 (39-41) |
| **Data on CD diagnosis, from medical records** | | |
| ***Age at CD diagnosis,*** *median, (IQR)* | 5 (4; 6) | 9 (8; 12) |
| ***Biopsy-verified CD diagnosis,*** *n (%)* | 25 (35) | 38 (44) |
| ***HLA-type,*** *n (%)* |  |  |
| *HLADQ2.5/DQ2.5* | 10 (14) | 26 (30) |
| *HLADQ2.2/DQ2.2* | 2 (3) | 0 (0) |
| *HLADQ2.5/DQ2.2* | 9 (12) | 11(13) |
| *HLADQ2.5/X, DQ2.2/X, DQ8/X* | 48 (67) | 45 (52) |
| *HLADQ8/8* | 3 (4) | 4 (5) |
| **Patient background, questionnaire data** | | |
| ***Comorbidity****, n (%)* |  |  |
| *Thyroid disease* | 2 (3) | 2 (3) |
| *Type 1 diabetes* | 2 (3) | 2 (3) |
| ***Heredity****^3^****,*** *n (%)* |  |  |
| *CD* | 18 (30) | 18 (24) |
| *Thyroid disease/T1DM* | 16 (22) | 14 (16) |
| **Pre-and perinatal data, from questionnaires** |  |  |
| ***Cesarean section,*** *n (%)* | 11 (15) | 5 (6) |
| ***Admission to neonatal ward****, n (%)* | 3 (5) | 5 (6) |
| ***Mother’s conditions during pregnancy,*** *n (%)* |  |  |
| *Infectious disease^2^* | 8 (11) | 12 (14) |
| *Other conditions^3^* | 2 (3) | 3 (4) |
| ***Mother’s use of medication during pregnancy,*** *n (%)* | | |
| *Antibiotics, corticosteroids, vaccines* | 11 (5) | 8 (9) |
| *Others^4^* | 34 (47) | 42 (49) |
| ***Mother’s use of nicotine during pregnancy****, n (%)* | 3 (6) | 1 (4) |
|  |  |  |

Data from the Swedish PKU biobank, patient medical records, and questionnaires, displayed for CD cases stratified on sex. Data from medical records were not available in comparators due to ethical approvals.

Percentages are reported from available data. *Any* questionnaire data was available for n=153 CD cases (96%).

^1^ At sample retrieval from the PKU biobank

^2^ Gastroenteritis, urinary tract infection, pneumonia, and other infections treated with antibiotics.

^3^ Conditions not requiring antibiotics (e.g., preeclampsia)

^4^ Including antidepressants, anxiolytics, analgesics, hormones, antihypertensives, and other medications.

HLA= human leukocyte antigen, IQR=interquartile range

### **Table S3.** Association between immune cell profiles at birth in children with celiac disease (CD) and matched comparators, stratified analyses by sex.

|  | **CD cases**  geometric mean (SD) | **Comparators**  geometric mean (SD) | **Fold-change,**  mean (95% CI) | **p-value** | **Interaction p-value** |  |
| --- | --- | --- | --- | --- | --- | --- |
| **Markers of T-and B-lymphocyte output** | | | | | | |
| *n, observations* | 158 | 316 |  |  |  |  |
| **TRECs,** *number of copies* | | | | | | |
| *Girls* | 136 (1.6) | 137 (1.6) | 0.99 (0.88; 1.12) | 0.91 |  |  |
| *Boys* | 107 (1.7) | 119 (1.7) | 0.89 (0.76; 1.04) | 0.14 | 0.26 |  |
| **KRECs,** *number of copies* | | | | | | |
| *Girls* | 72 (1.7) | 68 (1.7) | 1.07 (0.94; 1.21) | 0.34 |  |  |
| *Boys* | 60 (1.8) | 59 (1.8) | 1.02 (0.87; 1.21) | 0.78 | 0.71 |  |
| **Epigenetic cell counting, lymphocyte subsets proportions (of total leukocyte population)** | | | | | | |
| **n,** *observations** | 150 | 150 |  |  |  |  |
| **CD3+,** % |  |  |  |  |  |  |
| *Girls* | 32.1 (1.3) | 33.8 (1.5) | 0.95 (0.84; 1.08) | 0.44 |  |  |
| *Boys* | 35.9 (1.5) | 34.1 (1.5) | 1.05 (0.94; 1.19) | 0.37 | 0.25 |  |
| **CD4+,** % |  |  |  |  |  |  |
| *Girls* | 22.9 (1.4) | 25.3 (1.4) | 0.91 (0.80; 1.03) | 0.15 |  |  |
| *Boys* | 26.8 (1.4) | 24.5 (1.4) | 1.09 (0.99; 1.21) | 0.09 | 0.03 |  |
| **memCD4,** % |  |  |  |  |  |  |
| *Girls* | 0.4 (6.0) | 0.4 (6.6) | 1.00 (0.50; 2.01) | 1.00 |  |  |
| *Boys* | 0.4 (3.9) | 0.3 (4.8) | 1.26 (0.80; 1.97) | 0.31 | 0.57 |  |
| **Treg,** % |  |  |  |  |  |  |
| *Girls* | 1.6 (1.7) | 1.6 (1.8) | 0.97 (0.79; 1.18) | 0.74 |  |  |
| *Boys* | 1.9 (1.6) | 1.9 (1.6) | 0.97 (0.84; 1.12) | 0.71 | 0.95 |  |
| **CD8+,** % |  |  |  |  |  |  |
| *Girls* | 8.9 (1.6) | 9.2 (1.6) | 0.96 (0.81; 1.15) | 0.68 |  |  |
| *Boys* | 9.2 (2.2) | 9.5 (1.7) | 0.97 (0.80; 1.19) | 0.79 | 0.94 |  |
| **B,** % |  |  |  |  |  |  |
| *Girls* | 5.3 (1.6) | 5.7 (1.7) | 0.92 (0.77; 1.10) | 0.39 |  |  |
| *Boys* | 5.8 (1.7) | 5.8 (1.7) | 1.01 (0.87; 1.18) | 0.86 | 0.44 |  |
| **NK,** % |  |  |  |  |  |  |
| *Girls* | 2.3 (1.9) | 2.5 (2.0) | 0.94 (0.74; 1.19) | 0.62 |  |  |
| *Boys* | 2.8 (1.8) | 2.7 (2.0) | 1.03 (0.85; 1.25) | 0.76 | 0.57 |  |
|  | | | | | | |

T-cell receptor excision circle (TREC) and kappa-deleting recombination excision circle (KREC) levels at birth, expressed as number of copies/punch and lymphocyte subsets, expressed as % of nucleated cells. All variables have been log-transformed to approximate a normal distribution before analyses. The geometrical mean is the anti-log of this mean, and the fold-change refers to the ratio between the groups.

*Numbers for each subset are displayed in Figure 2, manuscript.

CI=confidence interval, SD=standard deviation,

### **Table S4.** Association between immune cell profiles at birth in children with celiac disease (CD) and matched comparators, stratified analyses by age at diagnosis (≥7 years or younger).

|  | **CD cases**  geometric mean (SD) | **Comparators**  geometric mean (SD) | **Fold-change,**  mean (95% CI) | **p-value** | **Interaction p-value** |
| --- | --- | --- | --- | --- | --- |
| **Markers of T- and B-lymphocyte output** | | | | | |
| n, *observations* | 158 | 316 |  |  |  |
| **TRECs,** *number of copies* | | | | | |
| *<7 years* | 134 (1.6) | 127 (1.7) | 1.07 (0.92; 1.23) | 0.38 |  |
| *≥7 years* | 113 (1.8) | 132 (1.6) | 0.86 (0.74; 0.99) | 0.04 | 0.03 |
| **KRECs,** *number of copies* | | | | | |
| *<7 years* | 62 (1.9) | 62 (1.8) | 1.01 (0.85; 1.19) | 0.94 |  |
| *≥7 years* | 72 (1.6) | 66(1.7) | 1.08 (0.95; 1.24) | 0.24 | 0.49 |
| **Lymphocyte subset proportions (of total leukocyte population)** | | | | | |
| n, *observations** | 150 | 150 |  |  |  |
| **CD3+,** % |  |  |  |  |  |
| *<7 years* | 34.1 (1.5) | 31.2 (1.5) | 1.10 (0.97; 1.26) | 0.15 |  |
| *≥7 years* | 34.5 (1.4) | 36.6 (1.5) | 0.94 (0.84; 1.05) | 0.27 | 0.07 |
| **CD4+,** % |  |  |  |  |  |
| *<7 years* | 24.0 (1.4) | 22.6 (1.4) | 1.06 (0.94; 1.21) | 0.34 |  |
| *≥7 years* | 26.0 (1.3) | 26.8 (1.4) | 0.97 (0.88; 1.07) | 0.60 | 0.27 |
| **memCD4,** % |  |  |  |  |  |
| *<7 years* | 0.4 (5.2) | 0.4 (4.1) | 0.91 (0.53; 1.55) | 0.72 |  |
| *≥7 years* | 0.5 (4.3) | 0.3 (6.6) | 1.41 (0.82; 2.44) | 0.22 | 0.26 |
| **Treg,** % |  |  |  |  |  |
| *<7 years* | 1.6 (1.7) | 1.5 (1.6) | 1.06 (0.89; 1.26) | 0.54 |  |
| *≥7 years* | 1.9 (1.5) | 2.1 (1.7) | 0.91 (0.78; 1.06) | 0.21 | 0.20 |
| **CD8+,** % |  |  |  |  |  |
| *<7 years* | 9.3 (1.5) | 8.2 (1.7) | 1.13 (0.95; 1.33) | 0.17 |  |
| *≥7 years* | 8.9 (2.2) | 10.5 (1.5) | 0.85 (0.69; 1.05) | 0.13 | 0.05 |
| **B,** % |  |  |  |  |  |
| *<7 years* | 5.1 (1.7) | 5.5 (1.7) | 0.91 (0.76; 1.09) | 0.31 |  |
| *≥7 years* | 6.1 (1.6) | 5.9 (1.7) | 1.03 (0.89; 1.20) | 0.66 | 0.29 |
| **NK,** % |  |  |  |  |  |
| *<7 years* | 2.4 (1.8) | 2.3 (1.7) | 1.05 (0.87; 1.27) | 0.62 |  |
| *≥7 years* | 2.8 (1.9) | 2.9 (2.2) | 0.95 (0.76; 1.18) | 0.64 | 0.51 |
|  | | | | | |

TREC and KREC levels at birth, expressed as number of copies/punch, and lymphocyte subsets, expressed as % of nucleated cells. All variables have been log-transformed to approximate a normal distribution before analyses. The geometrical mean is the anti-log of this mean, and the fold-change refers to the ratio between the groups.

*Numbers for each subset are displayed in Figure 2, Manuscript.

CI=confidence interval, KREC=kappa-deleting recombination excision circle, SD=standard deviation, TREC=T-cell receptor excision circles.

##

### **Table S5.** Association between immune cell profiles at birth in children with celiac disease (CD), presented for children with high risk vs. low risk for celiac disease, based on Humane Leucocyte Antigen (HLA)

|  | **High risk HLA**  Median (Q1, Q3) | **Low risk HLA**  Median (Q1, Q3) | **p-value** |
| --- | --- | --- | --- |
| **Markers of T- and B-lymphocyte output** | | | |
| ***Observations****, n* | 58 | 100 |  |
| **TRECs,** *no. of copies* | 125 (89.7; 149.6) | 118.5 (93.3; 176.7) |  |
| **LogTRECs** | 4.83 (4.5; 5.01) | 4.77 (4.54; 5.17) | 0.16 |
| **KRECs** *no. of copies* | 70.9 (42.8; 91.4) | 65.3 (47; 103.8) |  |
| **LogKRECs** | 4.26 (3.76; 4.52) | 4.18 (3.85; 4.64) | 0.78 |
| **Lymphocyte subset proportions (of total leukocyte population)** | | | |
| ***Observations,*** *n* | 56 | 94 |  |
| **CD3+,** % | 35.7 (27.9; 46.8) | 30.8 (26.7; 42.8) |  |
| **Log CD3** | 3.58 (3.33; 3.84) | 3.43 (3.29; 3.76) | 0.056 |
| **CD4+,**% | 26.5 (21.5; 31.3) | 24.5 (19.9; 29.2) |  |
| **LogCD4+** | 3.28 (3.09; 3.44) | 3.2 (2.99; 3.37) | 0.13 |
| **memCD4+,**% | 0.594 (0.166; 1.199) | 0.478 (0.173; 0.941) |  |
| **LogmemCD4+** | -0.435 (-1.519; 0.187) | -0.729 (-1.657; -0.061) | 0.55 |
| **CD8+,**% | 10.2 (6.9; 13) | 9.8 (7.2; 12.4) |  |
| **Log CD8+** | 2.32 (1.94; 2.56) | 2.28 (1.97; 2.52) | 0.54 |
| **Treg,** % | 1.99 (1.28; 2.67) | 1.56 (1.24; 2.18) |  |
| **LogTreg** | 0.688 (0.249; 0.984) | 0.443 (0.215; 0.778) | 0.12 |
| **B,** % | 5.68 (4.13; 8.22) | 5.42 (4.08; 8.11) |  |
| **LogB** | 1.74 (1.42; 2.11) | 1.69 (1.41; 2.09) | 0.63 |
| **NK,** % | 2.83 (1.9; 4.11) | 2.35 (1.55; 3.46) |  |
| **LogNK** | 1.04 (0.64; 1.41) | 0.855 (0.439; 1.243) | 0.061 |

TREC and KREC levels at birth, expressed as number of copies/punch, and lymphocyte subsets, expressed as % of nucleated cells**.**

Individuals homozygous for high-risk alleles encoding HLA-DQ2.5 (HLA-DQA1*05 and HLA-DQB1*02) or HLA-DQ2.2 (HLA-DQA1*02 and HLA-DQB1*02), as well as those carrying a combination of these (heterozygous for HLA-DQ2.5 and HLA-DQ2.2), were classified as the high-risk group. This group was compared with a low-risk group, which included individuals heterozygous for HLA-DQ2.5, HLA-DQ2.2, or carrying HLA-DQ8 (HLA-DQA1*03 and HLA-DQB1*03:02). A t-test was used to compare log-transformed continuous variables between the groups.

### **FIGURES**

### **Figure S1** Schematic overview of the measured immune cell markers in peripheral blood.


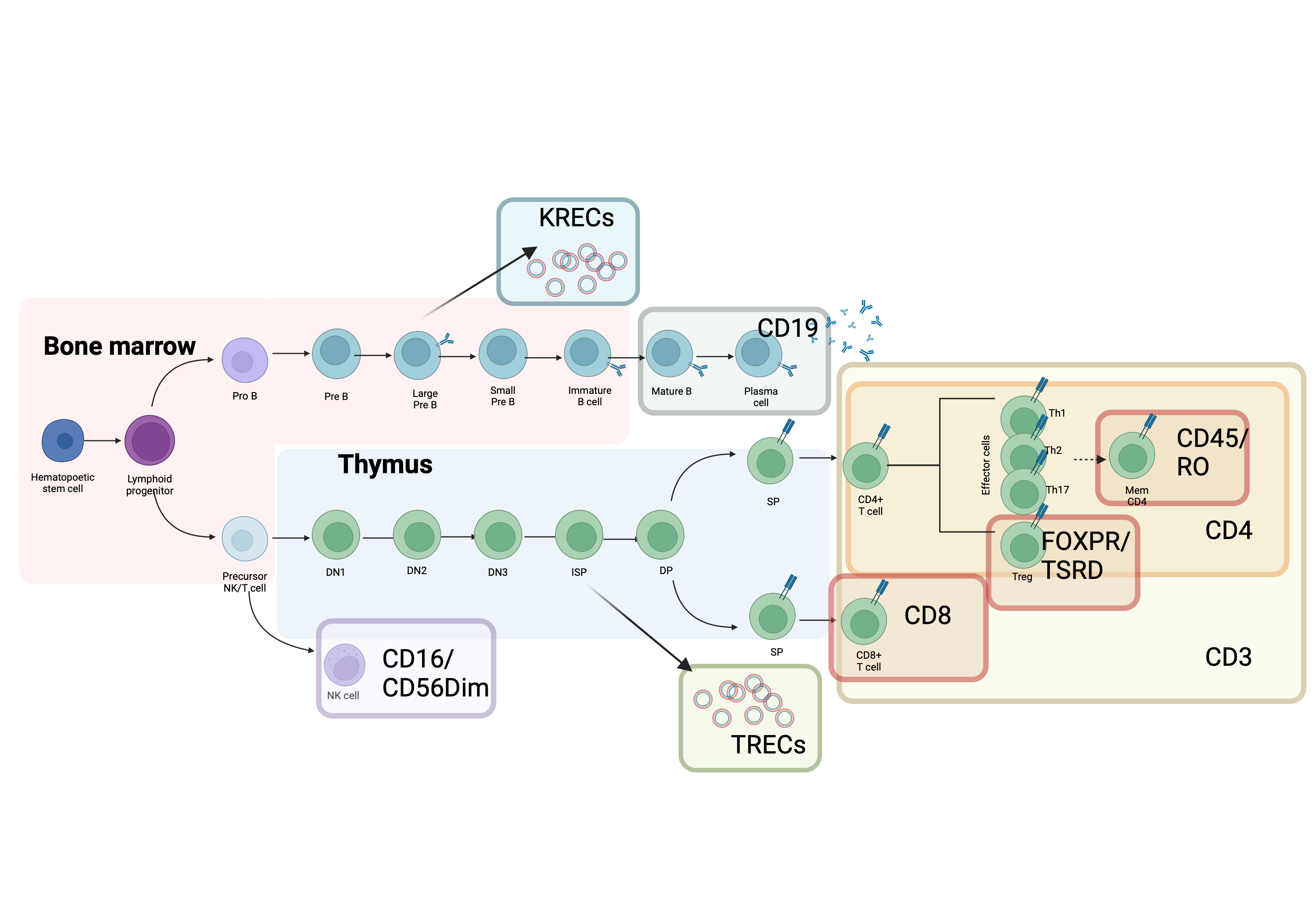


**Fig. S2** A simplified overview of the development of lymphocytes, from the hematopoietic stem cells, through the central tolerance process and formation of the subsets of lymphocytes. The colored boxes represent the immune cell markers in peripheral blood.

T cell formation begins in the bone marrow, where hematopoietic stem cells differentiate into lymphoid progenitors. These may migrate to the thymus. In the thymic cortex, progenitor cells commit to the T cell lineage and progress through the double-negative (DN) stages, marked by T cell receptor (TCR) rearrangement. During TCRβ rearrangement, excised DNA fragments form T cell receptor excision circles (TRECs), which serve as markers of recent thymic emigrants. Successfully rearranged TCRβ chains pair with pre-Tα to form the pre-TCR complex, driving proliferation and progression to the double-positive (DP) stage. In the thymic cortex, positive selection ensures recognition of self-MHC, and CD4/CD8 commitment depending on MHC restriction, while negative selection in the medulla eliminates autoreactive T cells, establishing central tolerance. Surviving cells mature into single-positive (SP) CD4+ or CD8+ T cells. Mature naïve T cells exit the thymus, carrying TRECs, and enter the periphery. During T cell development, CD3 is expressed as part of the TCR complex, while mature T cells upregulate activation markers such as CD45RO and transcription factors like FOXP3 (in regulatory T cells) to mediate their specific immune functions. (2)

B cells develop in the bone marrow, where progenitor cells rearrange their immunoglobulin genes. During immunoglobulin light chain rearrangement, excised DNA fragments form kappa-deleting recombination excision circles (KRECs), serving as markers of recent bone marrow emigrants. Successfully rearranged B cells express a functional B cell receptor (BCR) and undergo selection to ensure central tolerance. Mature naïve B cells then exit to the periphery. Created with BioRender.

**Figure S2** Flowchart of the formation of the study sample

**Fig. S1** Out of 162 celiac children in a previously described regional celiac cohort,(3) all but two newborn screening cards were available in the Swedish phenylketonuria (PKU) biobank and matched with two comparators (n=320). For KREC and TREC, we ran qPCR for all CD cases (n=160) and two comparators (n=320), and for epigenetic cell counting, all CD cases (n=160) and one comparator (160). Only successful qPCR results were included, and statistical comparisons only considered complete sets of CD cases: comparators, thus excluding CD cases and comparators where data lacked for the counterpart, thus data is presented for 158 CD cases and 316 comparators (98.8%) for KREC and TREC, and for 150 CD cases and 150 comparators (93.7%) for epigenetic cell counting. The specific number of observations for each lymphocyte subset is presented in Figure 2 (Manuscript)

### **Figure S3** Correlation between TRECs and CD3%


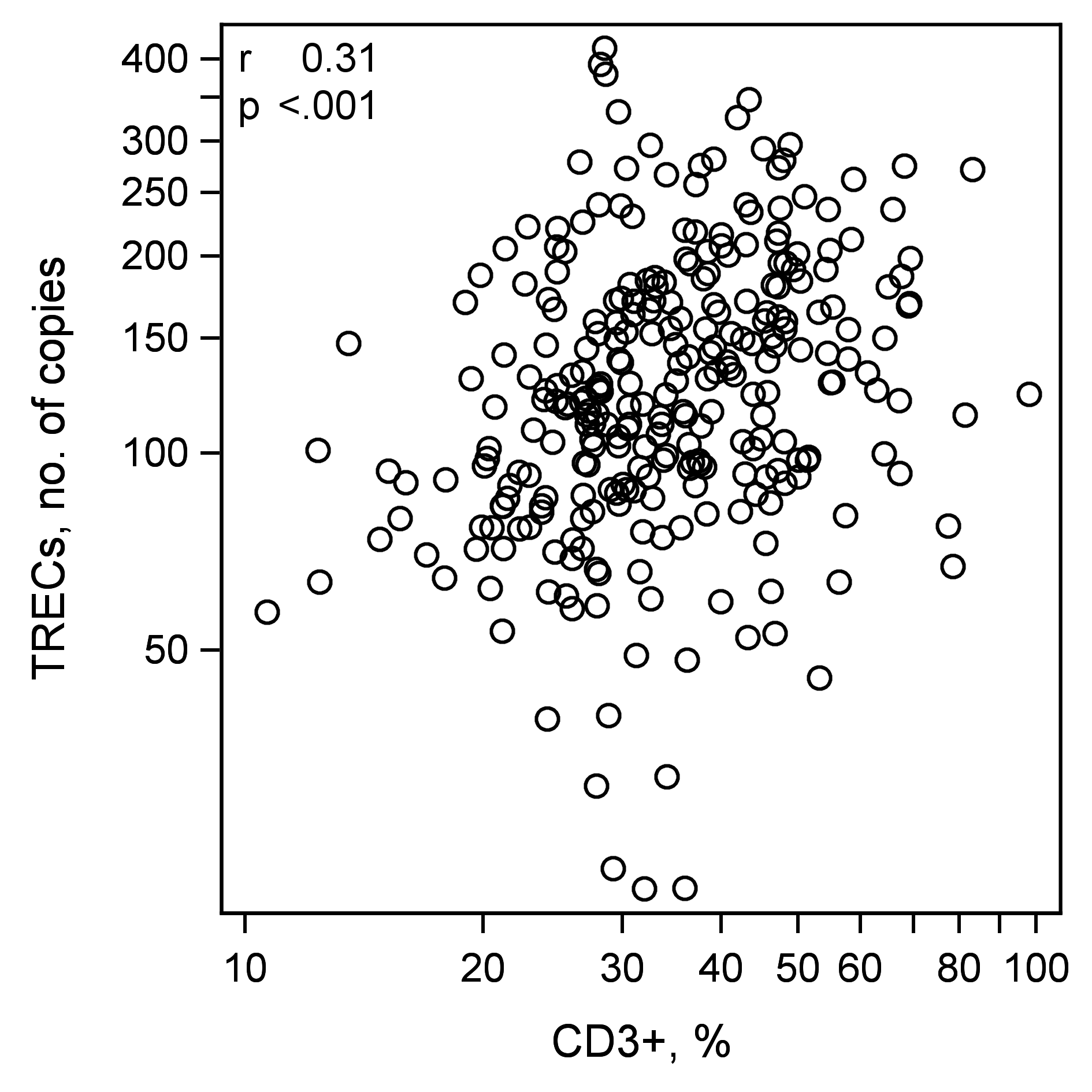


Spearman Rank Correlation for T cell receptor excision circles (TRECs), no. of copies/stance and demethylated copies of CD3+, % of total nucleated cells in the sample, in newborn children who later developed celiac disease, and from the general population.

### **Figure S4** Correlation between KRECs and B cells


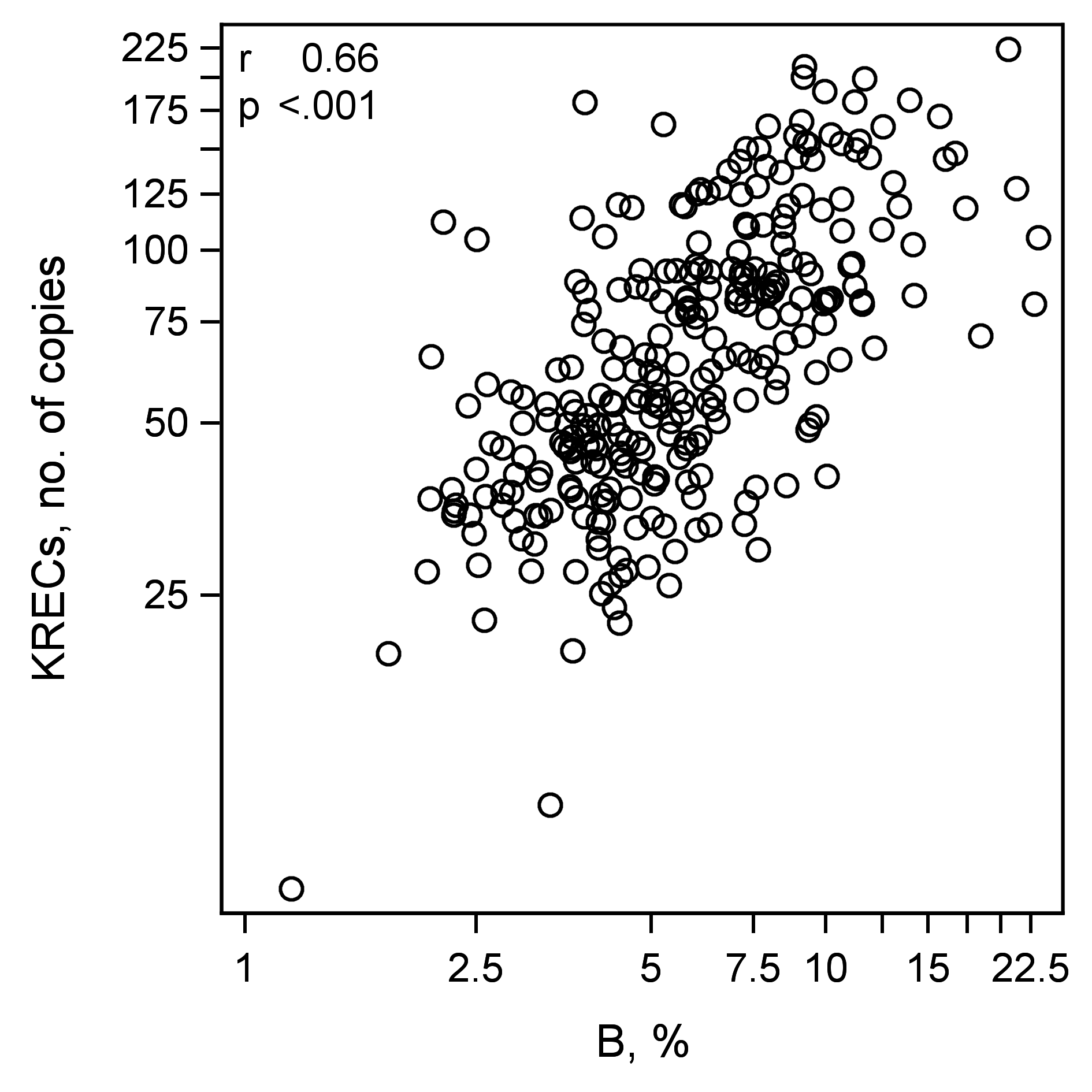


Kappa-deleting recombination excision circles (KRECs), no. of copies/stance and demethylated copies of CD19+ (B cells), % of total nucleated cells in the sample, in newborn children who later developed celiac disease, and comparators from the general population. Spearman Rank Correlation was used.

## **REFERENCES**

1 Gudmundsdottir JA, Thorgeirsdottir S, Lundback V, et al. Normal neonatal TREC and KREC levels in early onset juvenile idiopathic arthritis. Clin Immunol 2023;249(109277.

2 Ashby KM, Hogquist KA A guide to thymic selection of T cells. Nat Rev Immunol 2024;24(2):103-17.

3 Ulnes M, Albrektsson H, Stordal K, et al. Regional Swedish study found that one in seven coeliac patients experienced loss of follow up during childhood. Acta Paediatr 2022.
